# Detecting of Barely Visible Impact Damage on Carbon Fiber Reinforced Polymer Using Diffusion Ultrasonic Improved by Time-Frequency Domain Disturbance Sensitive Zone

**DOI:** 10.3390/s24103201

**Published:** 2024-05-17

**Authors:** Yuqi Ma, Fangyuan Li, Jianbo Wu, Zhaoting Liu, Hui Xia, Zhaoyuan Xu

**Affiliations:** School of Mechanical Engineering, Sichuan University, Chengdu 610065, China; yuqima@stu.scu.edu.cn (Y.M.); lifangyuan@scu.edu.cn (F.L.); liuzhaoting@stu.scu.edu.cn (Z.L.); xh@scu.edu.cn (H.X.); xuzhaoyuan@stu.scu.edu.cn (Z.X.)

**Keywords:** diffusion ultrasound, BVID, CFRP, nondestructive detection

## Abstract

Based on the decorrelation calculation of diffusion ultrasound in time-frequency domain, this paper discusses the repeatability and potential significance of Disturbance Sensitive Zone (DSZ) in time-frequency domain. The experimental study of Barely Visible Impact Damage (BVID) on Carbon Fiber Reinforced Polymer (CFRP) is carried out. The decorrelation coefficients of time, frequency, and time-frequency domains and DSZ are calculated and compared. It has been observed that the sensitivity of the scattered wave disturbance caused by impact damage is non-uniformly distributed in both the time and frequency domains. This is evident from the non-uniform distribution of the decorrelation coefficient in time-domain and frequency-domain decorrelation calculations. Further, the decorrelation calculation in the time-frequency domain can show the distribution of the sensitivity of the scattered wave disturbance in the time domain and frequency domain. The decorrelation coefficients in time, frequency, and time-frequency domains increase monotonically with the number of impacts. In addition, in the time-frequency domain decorrelation calculation results, stable and repetitive DSZ are observed, which means that the specific frequency component of the scattered wave is extremely sensitive to the damage evolution of the impact region at a specific time. Finally, the DSZ obtained from the first 15 impacts is used to improve the decorrelation calculation in the 16-th to 20-th impact. The results show that the increment rate of the improved decorrelation coefficient is 10.22%. This study reveals that the diffusion ultrasonic decorrelation calculation improved by DSZ makes it feasible to evaluate early-stage damage caused by BVID.

## 1. Introduction

Diffuse waves in plate are guided waves resulting from multi-scattering of elastic waves in heterogeneous media, highly sensitive to any structural disturbances [1]. Evaluating the damage level is an effective method based on the decorrelation between the disturbance signal and the reference signal [2].

Many studies have discussed many scattering wave indicators to evaluate the damage level. Pomarède et al. [3] analyze changes in relative wave velocity and the correlation of signals between reference and damage states to detect microcracks in Carbon Fiber Reinforced Polymer (CFRP) caused by the four-point bending test. Wojtczak et al. [2] uses the decorrelation of the coda signal in the time domain and frequency domain to evaluate the damage of the concrete cube under splitting conditions. Gao et al. [4] performed disbond detection of an aeronautical honeycomb composite sandwich by calculating windowed cross-correlation in time domain and local power spectral density in frequency domain for direct wave and coda wave. Spytek et al. [1] used synthetic time-reversal of diffuse Lamb waves for the mean wavenumber estimation algorithm and used ultrasonic coda waves to perform damage imaging on aluminum and CFRP plates. However, the contribution of the vibration components at different frequencies is undetermined. Spalvier et al. [5] utilized various features extracted from the cross-correlation function of multiple scattering signals to monitor the stress state in concrete pillars. These features include signal energy, cross-correlation amplitude, cross-correlation time and cross-correlation symmetry. Liu et al. [6] used Taylor series expansion to perform low-time-consuming cross-correlation calculations to analyze concrete cylinders’ relative wave velocity changes under compression conditions. He et.al. [7] established a physics-based model for the relative velocity change of coda wave subject to the stress variation for multi-layer structures. Niederleithinger et al. [8] devised a step-wise coda wave interferometry method for tracking stress change and distribution in concrete beams. Her et al. [9] uses the normalized coda wave energy of a single piezoelectric ceramic transducer to monitor the bolt connection. Furthermore, mode conversion [10] can also be used as damage indicators to evaluate structural integrity.

The scattering wave has different sensitivity to different positions on the specimen at different times [11]. The scattering wave sensitive kernel model can be used to estimate the distribution of sensitive areas in time domain and space domain [12]. The defect detecting and imaging can be realized by combining the decorrelation of signals between reference and damage states with the scattering wave sensitive kernel model [13,14]. However, it is difficult to establish a sensitive kernel model for a small-sized heterogeneous specimen, as the real fiber distribution is affected by processing, making it hard to obtain a complete multiple scattering model [15].

Impact damage in CFRP usually forms inside, including intra-layer matrix cracking, inter-layer cracking and fiber breakage [16,17]. Impact damage on composites is commonly referred to as Barely Visible Impact Damage (BVID) [18]. As damage accumulates, the stiffness of CFRP decreases and this degradation occurs in three stages. Initially, there is a rapid stiffness decrease due to matrix cracks, followed by a more gradual and slower degradation that typically accounts for the majority of the fatigue life. In the last part of the fatigue life, the material properties are drastically reduced and the stiffness loss is accelerated [19,20]. Currently, there is a lack of research on the distribution of sensitive areas of scattered waves in the time-frequency domain and the use of a Disturbance Sensitive Zone (DSZ) to improve detection sensitivity.

In this paper, the impact fatigue damage on CFRP is taken as the research object, and the change of time-frequency domain decorrelation of scattering wave under different impact times is discussed. The repeatability of time-frequency domain DSZ of scattering wave and the possibility of improving subsequent damage monitoring are also discussed. The improved decorrelation calculation results are compared with the decorrelation calculation results in time domain, frequency domain and time-frequency domain. This work will provide an experimental basis for the evaluation of the BVID based on scattering wave time-frequency domain decorrelation calculation methods improved by DSZ.

The rest of this paper is organized as follows. Section 2 introduces the experimental steps and the specimens used. Section 3 analyzes the experimental results, compares the decorrelation DC in time domain, frequency domain and time-frequency domain, and verifies the feasibility of using a prior DSZ to improve DC in the time-frequency domain. Finally, Section 4 concludes the study.

## 2. Materials and Methods

**Figure 1:**
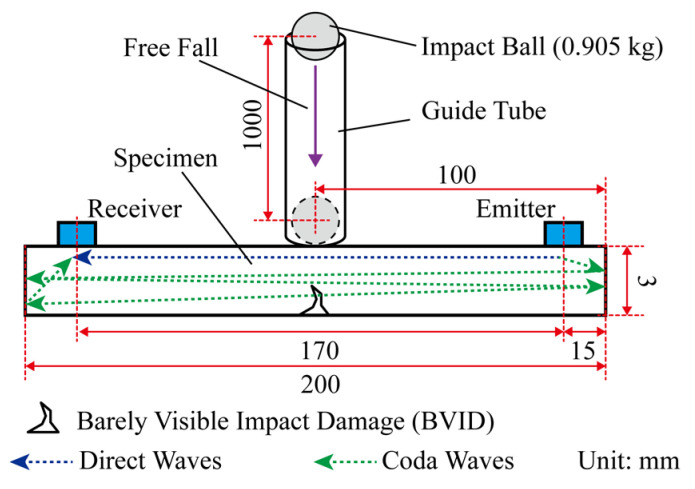
Impact process and diffusion ultrasonic propagation path.

**Figure 2:**
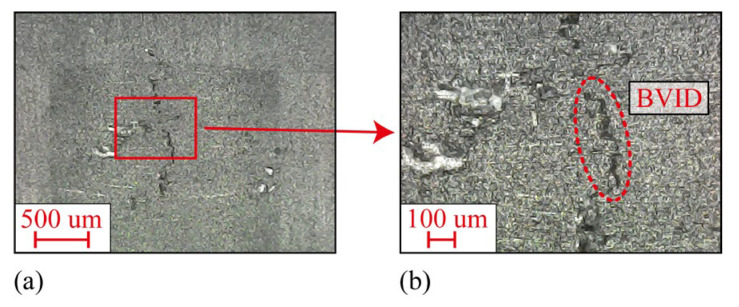
BVID was observed under an optical microscope. (**a**) Low magnification observation. (**b**) High magnification observation.

In order to produce different level of impact fatigue damage in specimen, a stainless steel iron ball with a mass of *m* = 0.905 kg and a diameter of *D* = 60 mm was used to impact the specimen. It is feasible to use ball to impact the specimen to produce different level of impact fatigue damage [21]. Single impact energy greater than 8 J. The specimen is CFRP with a size of 200 × 40 × 3 mm. The main properties of the specimen are shown in Table 1, and the supplier provides these property values. The impact ball falls freely from a height of *H* = 1000 mm and moves away quickly after it bounces up. During each impact process, only one contact occurs between the specimen and the impact ball. The impact process was carried out in a PVC guide tube with a length of *L* = 1000 mm and an inner diameter of *D*_pipe_ = 66 mm. The schematic diagram of the impact process and the diffusion ultrasonic propagation path is shown in Figure 1. The impact region is located in the center of the specimen. There is no obvious impact pit and damage on the surface of the impact region, and there is BVID on the back of the impact region. BVID was observed by X4D-Z03B042-D 1600× optical microscope (OM) produced by RIEVBCAU, as shown in Figure 2.

**Figure 3:**
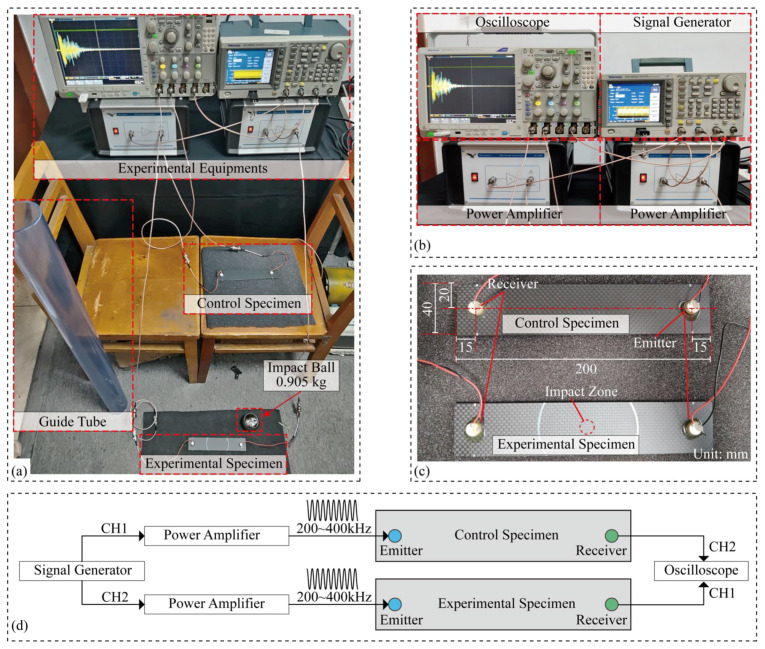
(**a**) Layout diagram. (**b**) Equipment wiring diagram. (**c**) Specimen connection diagram. (**d**) Wiring overview diagram.

The setup of the experiment is shown in Figure 3a, and the equipment wiring is shown in Figure 3b. Two PZT5A piezoelectric ceramics with a diameter of 10 mm and a thickness of 4 mm were fixed on the specimen using 801 chloroprene glue (AILIKE/801), as shown in Figure 3c. The wiring of the experiment process is shown in Figure 3d. A signal generator (Tektronix AFG3052C, Beverton, OR, USA) is used to generate a sweep signal of 200~400 kHz with a duration of 0.4 ms. The sweep signal is amplified by a power amplifier (Falco Systems WMA-300, Katwijk aan Zee, The Netherlands) and connected to the emitter probe. The receiver probe is connected to the oscilloscope (Tektronix MDO4034C). The signal is collected at a sampling rate of 500 MHz and filtered by an average of 128 times to remove the influence of random noise. The control specimen did not carry out the impact test, but the other steps were consistent with the experimental specimen, and the signal of the receiving probe was collected synchronously with the experimental specimen. A total of 20 impact tests were carried out. The ambient temperature was between 20.8 °C and 21.2 °C during the first 15 impact tests. During the 16-th to 20-th impact tests, the ambient temperature was between 20.6 °C and 20.8 °C. The change of ambient temperature is small, so its influence can be excluded.

## 3. Results

**Figure 4:**
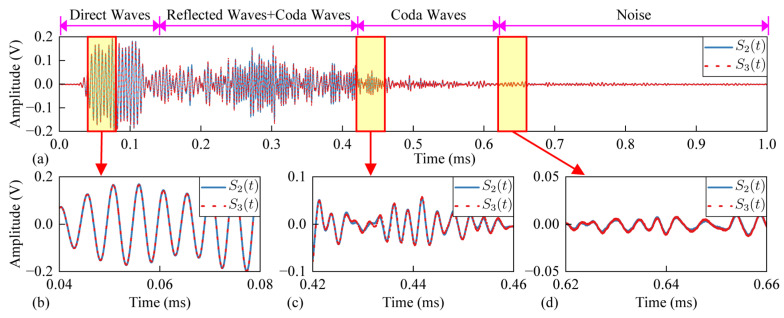
Collected signal (*S*_2_(*t*) is the signal of 2-th impact, *S*_3_(*t*) is the signal of 3-th impact). (**a**) Complete signal. (**b**) Direct wave. (**c**) Coda wave. (**d**) Noise.

The signal collected by the receiver probe is shown in Figure 4. According to the propagation time, the signal can be divided into direct wave, coda wave (multiply scattered wave) and noise. The ultrasonic wave attenuates rapidly when propagating in CFRP, and the coda wave is very short. Therefore, the decorrelation calculation of the signal part before noise (0~0.62 ms) is considered.

**Figure 5:**
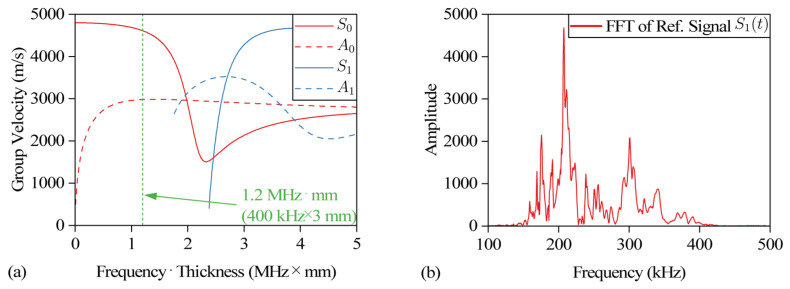
(**a**) Dispersion curve. (**b**) Spectrum diagram of signal S_1_(*t*).

The wavelength of the ultrasonic signal is in the same order of magnitude as the thickness of the specimen. The shear wave and the longitudinal wave will be reflected and superimposed between the upper and lower surfaces to form a special stress wave, namely Lamb wave. Carbon fiberboard is an anisotropic composite material. The speed of the ultrasonic wave propagating inside carbon fiberboard is related to the direction, and its true dispersion curve is complex [22]. Taking the shear wave velocity of 3 km/s and the longitudinal wave velocity of 5 km/s as examples, the dispersion curve of the isotropic plate with a thickness of 3 mm is drawn as shown in Figure 5a. S0 and S1 represent the 0th and 1st order symmetric mode Lamb waves, A0 and A1 represent the 0th and 1st order antisymmetric mode Lamb waves. The propagation velocity of the Lamb wave changes with the change in the frequency-thickness product. In this paper, Figure 5a is only used to illustrate the dispersion characteristics of Lamb propagation, which is not the real dispersion curve of ultrasonic wave in carbon fiber plate. The distribution of the disturbance sensitive zone in the time-frequency domain is related to the dispersion of Lamb, impact damage location and the distance between transducers, etc. The spectrum of S_1_(*t*) is shown as Figure 5b. Multiple peaks can be observed in the figure and they are related to the resonant frequency of the piezoelectric ceramic and specimen. The frequency range of the excitation signal is 200~400 kHz, so according to Figure 5a, we can assume that the expected modes are A0 and S0, where direct waves could be respectively S0 at 0.03~0.04 ms and A0 at about 0.06 ms. The second part of the signal mainly consists of the reflected waves (S0, A0) on edges and coda waves. The frontier between the second and third parts is more difficult to explain, but in the end of the signal we could supposed that there are mostly scattered waves. So we could supposed the third part as “Coda Waves”.

### 3.1. Time Domain Decorrelation

**Figure 6:**
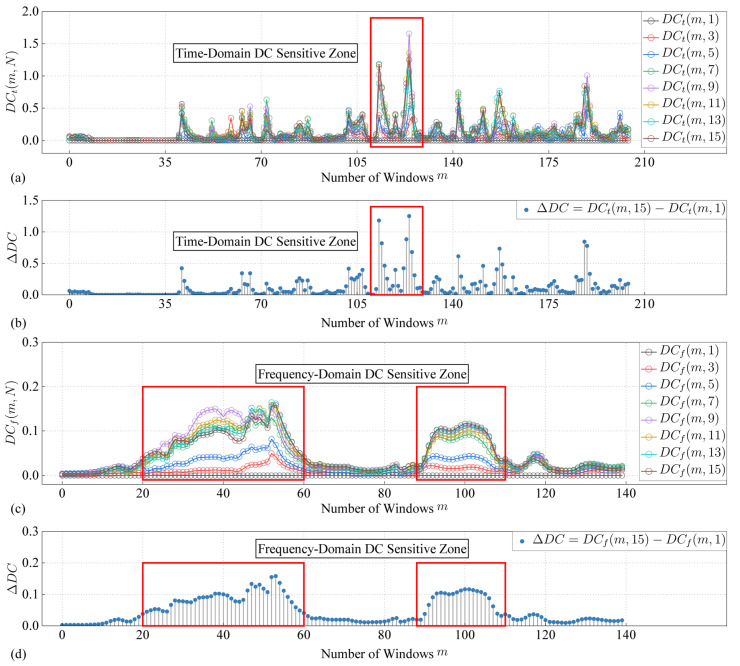
(**a**)Time domain *DC_t_*(*m*,*N*). (**b**) *DC_t_*(*m*,15) − *DC_t_*(*m*,1). (**c**) Frequency domain *DC_f_*(*m*,*N*). (**d**) *DC_f_*(*m*,15) − *DC_f_*(*m*,1).

The decorrelation calculation method of coda wave interferometry is used to calculate the collected signals. The reference signal is the signal *S*_1_(*t*) corresponding to the first impact, and the disturbance signal is the signal *S_N_*(*t*) corresponding to the *N*-th impact. In the time domain, the decorrelation coefficient *DC_t_*(*m*,*N*) of the *m*-th window of the *N*-th impact is calculated as follows:

(1) $$DC_{t}(m,N)=1-\frac{\int_{t_{m}}^{t_{m}+T_{W}}S_{1}(t)S_{N}(t)dt}{\sqrt{\int_{t_{m}}^{t_{m}+T_{W}}S_{1}^{2}(t)dt\int_{t_{m}}^{t_{m}+T_{W}}S_{N}^{2}(t)dt}}$$

where *DC_t_*(*m*,*N*) is the decorrelation coefficient corresponding to the *m*-th window of the N-th impact in the time domain. *t*_m_ is the starting time corresponding to the *m*-th window and *t*_0_ = 0, *T*_W_ = 6 us is the window length, the window overlap rate *O* = 50%, and the time domain calculation range is between 0~0.62 ms. The *DC* distribution in the time domain is shown in Figure 6a, where Figure 6b is the result of *DC_t_*(*m*,15) − *DC_t_*(*m*,1).

### 3.2. Frequency Domain Decorrelation

The Fourier transform of the signal is as follows:

(2) $$X_{N}(f)=\int_{0}^{T_{e}}S_{N}(t)e^{-iwt}dt$$

The end point of calculation *T_e_* = 0.8 ms. *X_N_*(*f*) is the spectrum of the signal corresponding to the *N*-th impact. In the frequency domain, the decorrelation coefficient *DC_f_*(*m*,*N*) of the *m*-th window of the *N*-th impact is calculated as follows:

(3) $$DC_{f}(m,N)=1-\frac{\int_{f_{m}}^{f_{m}+f_{W}}X_{1}(f)X_{N}(f)df}{\sqrt{\int_{f_{m}}^{f_{m}+f_{W}}X_{1}^{2}(f)df\int_{f_{m}}^{f_{m}+f_{W}}X_{N}^{2}(f)df}}$$

*f_m_* is the starting frequency corresponding to the *m*-th window and *f*_0_ = 200 kHz, *f*_W_ = 25 kHz is the window length, the window overlap rate *O* = 95%, and the frequency domain calculation range is between 200~400 kHz. The *DC* distribution in the frequency domain is shown in Figure 6c, where Figure 6d is the result of *DC_f_*(*m*,15) − *DC_f_*(*m*,1).

It can be seen from Figure 6 that *DC* is not non-uniform distributed in both time domain and frequency domain, and there is a sensitive area where *DC* value rises rapidly. There are multiple discrete sensitive zones in the time domain *DC_t_*. There are two obvious sensitive zones in the frequency domain *DC_f_*, which are near 260 kHz and 350 kHz respectively. Many factors, such as damage location, probe position, resonant frequency of piezoelectric ceramics and specimens, etc cause the non-uniform distribution of DC in time domain and frequency domain.

### 3.3. Time-Frequency Domain Decorrelation

**Figure 7:**
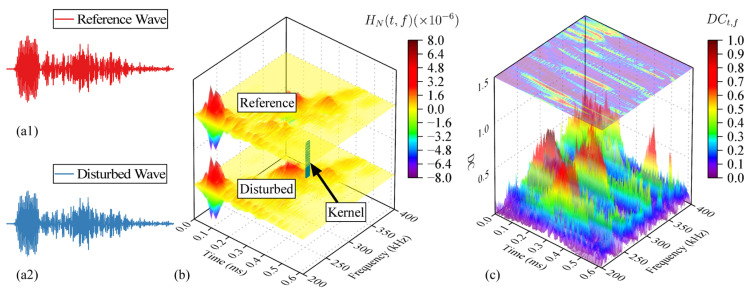
The calculation process of *DC_t_*_,*f*_, *N* = 2 in the figure. (**a1**) Reference signal (*S*_1_(*t*)). (**a2**) Perturbation signal (*S_N_*(*t*)). (**b**) Convolution process of *H*_1_(*t*,*f*) and *H_N_*(*t*,*f*). (**c**) *DC_t_*_,*f*_.

The short-time Fourier transform of the signal is as follows:

(4) $$F_{m,N}(f)=\int_{-\infty }^{\infty }S_{N}(t)g(t-mt_{s})e^{-j2\pi ft}dt$$

where *g*(*t* − *mt*_s_) is a rectangular sliding window with a length *T*_W_ = 500 us, and its position is determined by *mt_s_*. *t_s_* = 200 ns is the sliding step size, *m* = 1, 2, 3, …, k. F*_m_*_,*N*_(*t*,*f*) is the complex amplitude of the signal *S*_N_(t) between *t*_s_ and *t*_s_+ *T*_W_ on each frequency component. For the complex value *F_m_*_,*N*_(*t*,*f*) of the frequency component *f* at time *t*, the form is *F_m_*_,*N*_(*t*,*f*) = a + bi, absolute value of amplitude *A* = sqrt(a^2^ + b^2^), phase *p* = arctan(b/a). Therefore, the amplitude of each frequency component is restored as follows:

(5) $$H_{N}(t,f)=\frac{2A}{N_{sum}}\cos(2\pi ft+P)$$

where *N_sum_* is the total number of sampling points, and *H_N_*(*t*,*f*) is the *N*-th impact signal amplitude of the frequency component *f* at time *t*. The calculation of decorrelation *DC_t_*_,*f*_ in time-frequency domain is shown in Figure 7. The short-time Fourier transform and amplitude conversion of the reference signal *S*_1_(*t*) (Figure 7a1) and the disturbance signal *S_N_*(*t*) (Figure 7a2) are performed to obtain *H*_1_(*t*,*f*) and *H_N_*(*t*,*f*) as shown in Figure 7b. The time-frequency domain decorrelation *DC_t_*_,*f*_ is calculated by a kernel as follows:

(6) $$DC_{t,f}=1-\frac{\int_{f-f_{h}}^{f+f_{h}}\int_{t-t_{h}}^{t+t_{h}}H_{1}(t,f)H_{N}(t,f)dtdf}{\sqrt{\int_{f-f_{h}}^{f+f_{h}}\int_{t-t_{h}}^{t+t_{h}}H_{1}^{2}(t,f)dtdf\int_{f-f_{h}}^{f+f_{h}}\int_{t-t_{h}}^{t+t_{h}}H_{N}^{2}(t,f)dtdf}}$$

where *t_h_* = 2 us is half of the length in the kernel time axis direction, and *f_h_* = 2 kHz is half of the length in the kernel frequency axis direction. The calculated *DC_t_*_,*f*_ is shown in Figure 7c.

### 3.4. Disturbance Sensitive Zone

**Figure 8:**
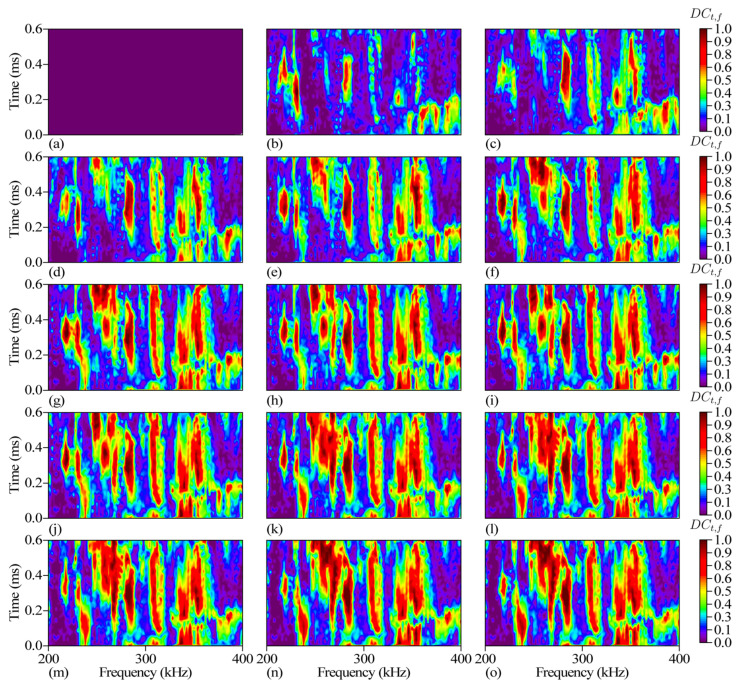
(**a**) *DC_t_*_,*f*_ of the 1-st impact. (**b**) *DC_t_*_,*f*_ of the 2-nd impact. (**c**) *DC_t_*_,*f*_ of the 3-rd impact. (**d**) *DC_t_*_,*f*_ of the 4-th impact. (**e**) *DC_t_*_,*f*_ of the 5-th impact. (**f**) *DC_t_*_,*f*_ of the 6-th impact. (**g**) *DC_t_*_,*f*_ of the 7-th impact. (**h**) *DC_t_*_,*f*_ of the 8-th impact. (**i**) *DC_t_*_,*f*_ of the 9-th impact. (**j**) *DC_t_*_,*f*_ of the 10-th impact. (**k**) *DC_t_*_,*f*_ of the 11-th impact. (**l**) *DC_t_*_,*f*_ of the 12-th impact. (**m**) *DC_t_*_,*f*_ of the 13-th impact. (**n**) *DC_t_*_,*f*_ of the 14-th impact. (**o**) *DC_t_*_,*f*_ of the 15-th impact.

**Figure 9:**
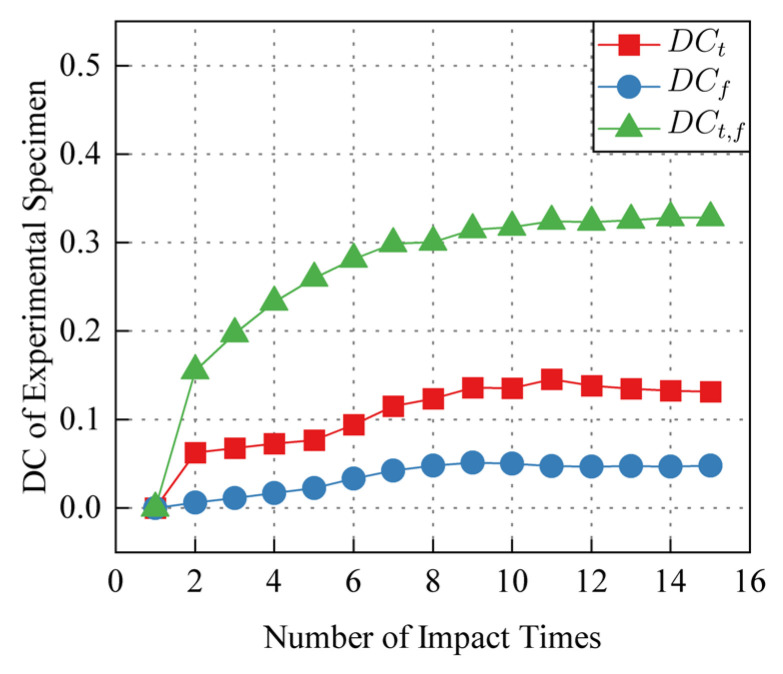
Experimental specimen *DC* in time domain, frequency domain and time-frequency domain.

**Figure 10:**
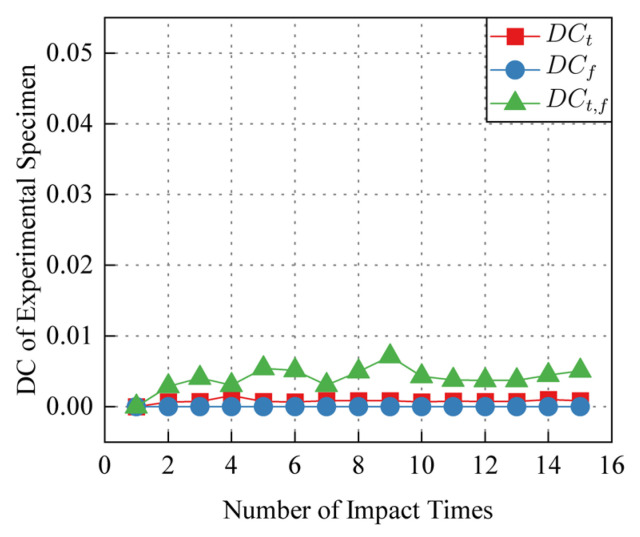
Control specimen *DC* in time domain, frequency domain and time-frequency domain.

Taking the signal of the 1-st impact as the reference signal, the *DC_t_*_,*f*_ of 1-st to 15-th impact is calculated according to the calculation process shown in Figure 7, and the results are shown in Figure 8. *DC_t_*_,*f*_ increases with the increase of the number of impact. The increase of *DC_t_*_,*f*_ is non-uniformly distributed in the time-frequency domain. *DC_t_*_,*f*_ rises rapidly in some regions, and the position of these regions in the time-frequency domain is relatively stable. Changes of time-domain decorrelation *DC_t_*, frequency-domain decorrelation *DC_f_*, time-frequency domain decorrelation *DC_t_*_,*f*_ with the number of impacts in the experimental and control specimens are shown in Figure 9 and Figure 10. Compared with *DC_t_* and *DC_f_*, *DC_t_*_,*f*_ is more sensitive to impact fatigue damage and can better evaluate the evolution of impact fatigue damage. In order to further discuss the region where *DC_t_*_,*f*_ rises rapidly in the time-frequency domain, the region that deviates from most values in *DC_t_*_,*f*_ is regarded as the DSZ.

**Figure 11:**
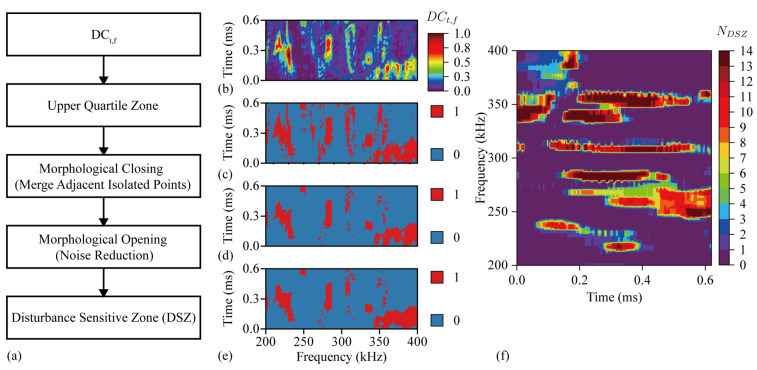
(**a**) *DSZ* calculation flow chart. (**b**) *DC_t_*_,*f*_. (**c**)The upper quartile region of *DC_t_*_,*f*_. (**d**) Result of morphological closed operation. (**e**) Result of morphological open operation. (**f**) The number of overlaps *N_DSZ_*.

The calculation flow chart of DSZ is shown in Figure 11a. The value of the upper quartile region deviates from the distribution of most values, which means that the *DC_t_*_,*f*_ in this region rises rapidly when the disturbance occurs. After the morphological closed operation and open operation of the upper quartile region, the DSZ is obtained. *DC_t_*_,*f*_ before processing is shown in Figure 11b,c is the upper quartile region of *DC_t_*_,*f*_, Figure 11d is the result of morphological closed operation of Figure 11c,e is the result of morphological open operation of Figure 11d. Figure 11c–e are binary graph, where the red area is the target area.

The *DSZ* of 2-nd to 15-th impact is superimposed, and the distribution of the number of overlaps *N_DSZ_* in the time-frequency domain is shown in Figure 11f. The region of *N_DSZ_* = 14 in the figure means that *DC_t_*_,*f*_ in these regions rises rapidly in all the disturbance signals from the 2-nd impact to the 15-th impact. These regions are stable and highly repeatable DSZs in the time-frequency domain.

It can be seen from Figure 6c that the sensitive region can be divided into two parts in the frequency domain. The disturbance-sensitive zone *DSZ*_l_ (frequency range 200~300 kHz, time 0~0.62 ms) and *DSZ*_h_ (frequency range 300~400 kHz, time 0~0.62 ms) were divided by 300 kHz as the dividing line for analysis. In order to further analyze the change of the distribution characteristics of *DSZ* with the increase of the number of impact, *LDSZ*(N) = (*CP_f_*, *CP_t_*, *N*) is used as the weighted average position of *DC_t_*_,*f*_ in the *DSZ* of the *N*-th impact. *CP_t_* and *CP_f_* are calculated as Equation (7) and Equation (8), respectively.

(7) $$CP_{f}=\frac{\int_{f_{s}}^{f_{e}}\int_{t_{s}}^{t_{e}}f\cdot DC_{t,f}dtdf}{\int_{f_{s}}^{f_{e}}\int_{t_{s}}^{t_{e}}DC_{t,f}dtdf}$$

(8) $$CP_{t}=\frac{\int_{t_{s}}^{t_{e}}\int_{f_{s}}^{f_{e}}t\cdot DC_{t,f}dfdt}{\int_{t_{s}}^{t_{e}}\int_{f_{s}}^{f_{e}}DC_{t,f}dfdt}$$

**Figure 12:**
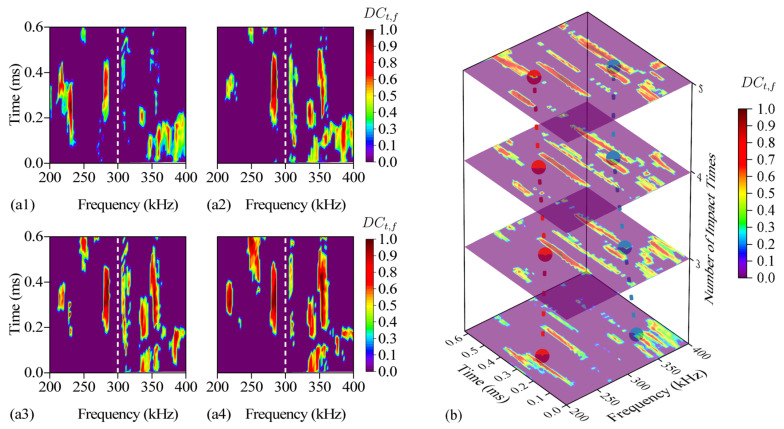
(**a1**) *DC_t_*_,*f*_|*DSZ* of the 2-nd impact. (**a2**) *DC_t_*_,*f*_|*DSZ* of the 3-rd impact. (**a3**) *DC_t_*_,*f*_|*DSZ* of the 4-th impact. (**a4**) *DC_t_*_,*f*_|*DSZ* of the 5-th impact. (**b**) The red line represents *LDSZ*_l_(N) and the blue line represents *LDSZ*_h_(N).

For *DSZ*_l_, *f_s_* = 200 kHz, *f_e_* = 300 kHz, *t_s_* = 0, *t_e_* = 0.62 ms. For *DSZ*_h_, *f_s_* = 300 kHz, *f_e_* = 400 kHz, *t_s_* = 0, *t_e_* = 0.62 ms. The weighted center positions of *DSZ*_l_ and *DSZ*_h_ are *LDSZ*_l_(N) and *LDSZ*_h_(N), respectively. The distribution of the 2-nd to 5-th impact of *LDSZ*_l_(N) and *LDSZ*_h_(N) is shown in Figure 12.

**Figure 13:**
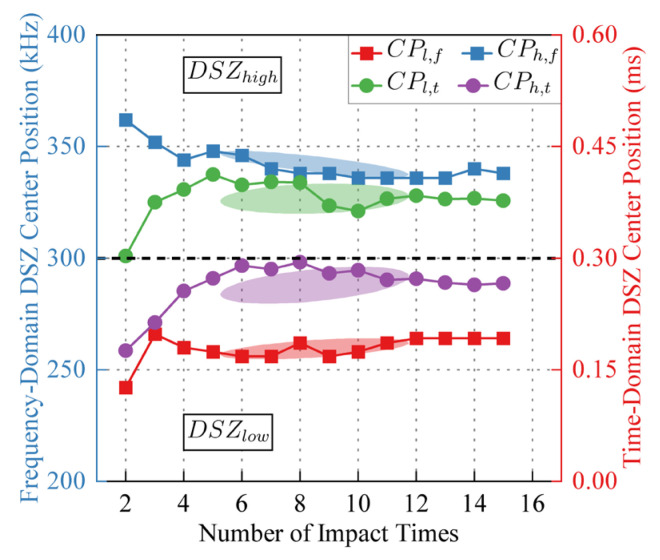
The projection of *LDSZ*(N) in time domain and frequency domain.

*LDSZ*(N) can characterize the distribution characteristics of *DC_t_*_,*f*_|DSZ in time-frequency domain. *LDSZ*_l_(N) and *LDSZ*_h_(N) are projected onto the time domain to obtain *CP*_l_(*t*) and *CP_h_*(*t*), and *LDSZ*_l_(N) and *LDSZ*_h_(N) are projected onto the frequency domain to obtain *CP*_l_(*f*) and *CP_h_*(*f*). The variations of *CP*_l_(*t*), *CP_h_*(*t*), *CP*_l_(*f*) and *CP_h_*(*f*) with the increase of the number of impact are shown in Figure 13, where the confidence level of confidence ellipse is 95%. A confidence ellipse can show the distribution of data points. As the correlation between the two variables increases, the confidence ellipse will be elongated toward greater correlation. The equation of the confidence ellipse of the variables *x* and *y* is shown as follows:

(9) $$\frac{{\left(x-\bar{x}\right)}^{2}}{\sigma_{x}^{2}}-2\rho \frac{\left(x-\bar{x})(y-\bar{y}\right)}{\sigma_{x}\sigma_{y}}+\frac{{\left(y-\bar{y}\right)}^{2}}{\sigma_{y}^{2}}=c$$

where $\bar{x}$ and $\bar{y}$ are the mean values of *x* and *y*, respectively, *σ_x_* and *σ_y_* are the standard deviations of *x* and *y*, and *ρ* is the correlation coefficient of *x* and *y*. *c* is the confidence level determined by the chi-square distribution, and *c* = 5.991 when the confidence interval is 95%. The confidence ellipse in this paper is drawn using Origin 2022 software.

Observe the scatters and confidence ellipses in Figure 13, as the number of impact increases, the *DSZ* shifts slightly backward in the time domain, which means that as the number of impact increases, the response of the signal part with longer propagation time to the disturbance is strengthened. As the number of impacts increases, *DSZ*_l_ approaches 260 kHz in the frequency domain, and DSZh approaches 350 kHz in the frequency domain, consistent with the distribution of decorrelation-sensitive areas in the frequency domain observed in Figure 6.

### 3.5. DC Improving by Prior DSZ

*DSZ*_2-15_ is the region in the DSZ of the 2-nd to 15-th impact that is stably repeated 14 times (stably repeated each DSZ), where *N*_DSZ_ = 14. The *DC_t_*_,*f*_ in DSZ_2-15_ is extremely sensitive to the damage evolution of the impact area. Therefore, the *DC_t_*_,*f*_ in DSZ is analyzed, where *DC_t_*_,*f*_|DSZ represents the *DC_t_*_,*f*_ value in DSZ.

**Figure 14:**
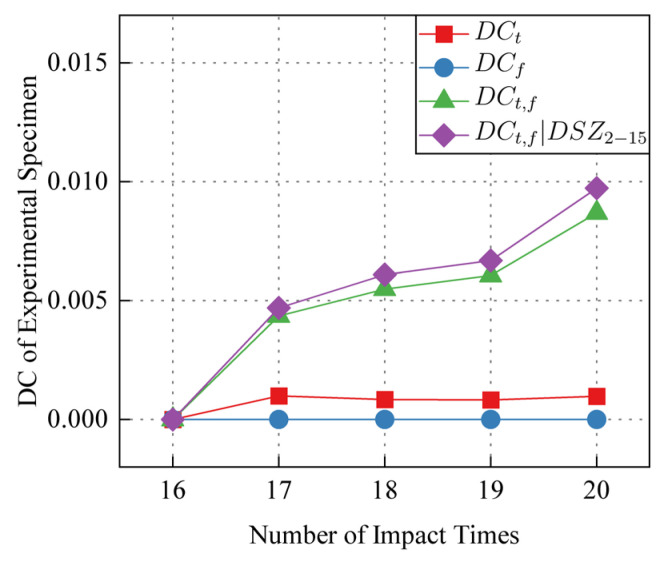
Time domain *DC_t_*, frequency domain *DC_f_*, time-frequency domain *DC_t_*_,*f*_, prior *DSZ* improved *DC_t_*_,*f*_|*DSZ*_2-15_.

The 16–20 th impact is the later stage of the continuous impact experiment. This part of the impact experiment can be used to discuss whether the *DSZ* obtained in the previous impact experiment can be used to improve the detection of the subsequent evolution of the impact damage. The impact fatigue damage on CFRP is divided into three stages [18]. The impact fatigue damage at the initial stage of life and after the life of 70% increases rapidly with the increase of the number of impact. The damage evolution in the second stage is gentle and not obvious. Therefore, the decorrelation between the 16-th and 20-th impact can be expected to change small. Taking the signal *S*_16_(*t*) of the 16-th impact as the reference signal, the calculation results of decorrelation *DC* for the signals of the 17-th to 20-th impact are as shown in Table 2. Time domain *DC_t_*, frequency domain *DC_f_*, time-frequency domain *DC_t_*_,*f*_, prior DSZ improved *DC_t_*_,*f*_|*DSZ*_2-15_ are as shown in Figure 14.

It can be seen from Figure 14 that the use of a priori stable and repeatable *DSZ* can further improve the monitoring of subsequent *DC* changes. The increase rate IR is calculated as follows:

(10) $$IR=\frac{\left.DC_{t,f}\right|DSZ_{1-15}-DC_{t,f}}{DC_{t,f}}$$

The results show that using the prior *DSZ* to improve the subsequent *DC* can obtain higher sensitivity, which is helpful in further detecting the evolution of impact fatigue damage on CFRP. The improved time-frequency domain *DC_t_*_,*f*_ increase rate is 10.22% on average.

## 4. Conclusions

The evaluation of impact fatigue damage on CFRP using scattering waves was studied. The scattered wave signals under different the number of impact are used as reference signals and disturbance signals. The time domain, frequency domain and time-frequency domain decorrelation calculations are performed to evaluate the evolution of impact damage. The distribution characteristics of the disturbance sensitive zone in the time-frequency domain and the feasibility of using the disturbance sensitive zone to improve the subsequent decorrelation calculation are discussed. The following conclusions are obtained:

(1) The *DC* in time domain, frequency domain and time-frequency domain increases with the increase of the number of impact, which indicates that *DC* in time domain, frequency domain and time-frequency domain can be used to evaluate the evolution of impact damage. In addition, the *DC* in the time-frequency domain shows higher sensitivity to the damage evolution of the impact region than the *DC* in the time domain and frequency domain.

(2) The sensitive region where *DC* rises rapidly is observed in both time domain and frequency domain. The sensitive region where *DC* rises rapidly can also be observed in the time-frequency domain, and its distribution characteristics LDSZ is consistent with those observed in the time domain and frequency domain.

(3) Based on the prior stable and highly repetitive disturbance sensitive zone, the decorrelation calculation of the time domain *DC_t_*, frequency domain *DC_f_*, time-frequency domain *DC_t_*_,*f*_ and the prior *DSZ* improved *DC_t_*_,*f*_|*DSZ*_2-15_ of the 16-th to 20-th impact signals is carried out. The results show that the prior *DSZ* can further improve the sensitivity of the time-frequency domain *DC* to the damage evolution of the impact region, and the average increase rate reaches 10.22%.

The research results of this paper show that there are disturbance-sensitive zones which are extremely sensitive to the damage evolution of the impact region and are stable and repeatable in the time-frequency domain of the scattered wave. Using these DSZ to improve the calculation of time-frequency domain decorrelation *DC_t_*_,*f*_ is helpful to study the evolution of impact fatigue damage on CFRP. Further research will be carried out on different types of composite materials in the future.

## Figures and Tables

**Table 1:** The main properties of the specimen.

Property	Specification
Model	T300
Number of fiber filaments	3 K
Filament Diameter	7 um
Density	1.76 g/cm^3^
Size	200 × 40 × 3 mm

**Table 2:** The calculation results of decorrelation *DC*.

Number of Impact	*DC_t_*	*DC_f_*	*DC_t_*_,*f*_	*DC_t_*_,*f*_|*DSZ*_2-15_	Increase Rate *IR*
17	9.88314 × 10^−4^	1.31597 × 10^−6^	0.00436	0.00469	7.5688%
18	8.40645 × 10^−4^	2.88991 × 10^−6^	0.00548	0.00609	11.1314%
19	8.26131 × 10^−4^	4.10172 × 10^−6^	0.00606	0.00668	10.2310%
20	9.84513 × 10^−4^	8.63983 × 10^−6^	0.00869	0.00973	11.9678%

## Data Availability

Data will be made available on request.
